# *Lacticaseibacillus rhamnosus* LRa05 in the treatment of acute diarrhea in children: a randomized controlled trial

**DOI:** 10.3389/fnut.2024.1479186

**Published:** 2024-11-15

**Authors:** Ke Chen, Kaihong Zeng, Shanshan Jin, Yu Ma, Limei Cai, Ping Xu, Yang Nie, Li Luo, Qinghua Yu, Changqi Liu

**Affiliations:** ^1^Department of Nutrition, Chengdu Women’s and Children’s Central Hospital, School of Medicine, University of Electronic Science and Technology of China, Chengdu, China; ^2^Department of Health Management Center, Institute of Health Management, Sichuan Provincial People’s Hospital, University of Electronic Science and Technology of China, Chengdu, China; ^3^Department of Nutrition, Chengdu Women’s and Children’s Central Hospital, School of Medicine, University of Electronic Science and Technology of China, Chengdu, China; ^4^Department of Neonatology, Dayi Maternal and Child Health Care Hospital, Chengdu, China; ^5^Department of Neonatology, Qingbaijiang Maternal and Child Health Care Hospital, Chengdu, China; ^6^Department of Child Health Care, Qingbaijiang Maternal and Child Health Care Hospital, Chengdu, China; ^7^Department of Child Health Care, Chongzhou Maternal and Child Health Care Hospital, Chengdu, China; ^8^Department of Pediatrics, Dayi Maternal and Child Health Care Hospital, Chengdu, China; ^9^Laboratory of Microbiology, Immunology, and Metabolism, DiPROBIO (Shanghai) Co., Limited, Shanghai, China; ^10^School of Exercise and Nutritional Sciences, San Diego State University, San Diego, CA, United States

**Keywords:** probiotic, diarrhea, children, gut microbiota, RCT

## Abstract

**Introduction:**

The goal of this study is to assess the efficacy and safety of *Lacticaseibacillus rhamnosus* LRa05, as an adjunct to the treatment of acute watery diarrhea in children.

**Methods:**

Eligible diarrheal children were randomized into intervention group (IG, *n* = 57) and control group (CG, *n* = 54), and given probiotics or placebo, respectively.

**Results:**

The total duration of diarrhea in the IG (121.4 ± 13.7 h) was significantly shorter than that in the CG (143.9 ± 19.8 h, *p* < 0.001). More children in the IG showed improvements in diarrhea than those in the CG for both per protocol analysis (70.2 vs. 46.3%, *p* = 0.01) and intention-to-treat analysis (66.7 vs. 41.7%, *p* = 0.003). The LL-37 levels in the IG was markedly higher than that in the CG after the intervention (4349.35 ± 1143.86 pg./g vs. 3682.49 ± 869.21 pg./g, *p* = 0.039). The intervention led to higher abundance of *Bifidobacterium longum* and lower abundance of *Enterococcus faecium*, *Lactobacillus rhamnosus*, and *Bacteroides fragilis* (*p* < 0.05). LRa05 treatment upregulated the functional genes of gut microbiota involving immunity regulation.

**Discussion:**

Administration of the *Lacticaseibacillus rhamnosus* LRa05 at a dose of 5 × 10^9^ CFU/day to children aged 0-3 years resulted in shorter duration of diarrhea, faster improvement in fecal consistency, and beneficial changes in gut microbiome composition and gene functions.

**Clinical trial registration:**

The present study has been approved and registered in the Chinese Clinical Trial Registration Center with the registration number of ChiCTR2100053700 (https://www.chictr.org.cn/showproj.html?proj=141082).

## Introduction

Diarrhea is a common and frequently occurring disease, which can cause malnutrition, restricted growth and development, and even child death, especially in developing countries (1). In China, children under the age of 5 years have a high incidence rate of diarrhea at 5.51% per year (95% CI: 3.76–8.01).

In recent years, probiotic supplementation has been proposed as a complement to the treatment of acute diarrhea. Currently, hundreds of different probiotic products are available in the market. These products differ in excipients, amount and strains of microorganisms, and their activity (2–5). However, the specific effects of probiotics are highly dependent on “strain specificity” (6). The European Society for Pediatric Gastroenterology, Hepatology, and Nutrition (ESPGHN) and the European Society of Pediatric Infectious Diseases Expert Working Group stated that only probiotic strains with proven clinical efficacy and in appropriate dosage may be recommended as an adjuvant to treat children with acute gastroenteritis (7).

*Lacticaseibacillus rhamnosus* LRa05 is a specific strain isolated from infant feces with independent intellectual property rights. The strain has been assigned a preservation number of CGMCC no. 24377 by the China General Microbiological Culture Collection Center (CGMCC).

To our knowledge, no study has investigated whether LRa05 can achieve good colonization and become a dominant flora to play its immune enhancing role in children with diarrhea. Therefore, the purpose of this research is to study the adjunctive clinical efficacy of the LRa05 strain on acute watery diarrhea of children.

## Materials and methods

### Subjects and ethical approval

This is a multi-center, parallel randomized, controlled, double-blinded clinical intervention. Children of both sexes and aged 0–3 years who were outpatients and/or hospitalized with diarrhea were recruited from December 2021 to September 2022.

*Inclusion, exclusion, and withdrawal criteria.*

Diagnostic criteria for watery diarrhea: Increased fecal frequency (≥ 4 times/day) (8) with watery feces (Bristol fecal score above type 6).

*Inclusion criteria:*

1. Age: children 0–3 years old.
2. Duration of diarrhea: more than 12 h and <72 h;
3. Diagnosed as non-bacterial infectious diarrhea when recruited;

*Exclusion criteria:*

1. Chronic and/or persistent diarrhea;
2. Nervous system dysplasia and severe organic diseases;
3. Moderate and severe dehydration and serious illnesses requiring Pediatric Intensive Care Unit (PICU) treatment;
4. Had taken the same probiotics within 1 month before the diagnosis of this illness;
5. Children expected to receive antibiotic treatment during the trial.

*Withdrawal criteria:*

1. Children without any clinical records for evaluation;
2. Children taking drugs prohibited by the study, including hormones, immunosuppressive drugs, other probiotics, etc., during the treatment;
3. Children whose condition worsened and needed to be admitted to PICU during the treatment.

### Grouping and intervention

A biostatistician, who was not directly involved in the execution of the study, used the RAND function in Excel to generate random numbers. Children who met the inclusion criteria were coded by the random numbers and assigned into the two groups based on the sequence of the random numbers. Each group was randomly assigned with 60 children.

Socio-demographic data were collected at baseline. Hydration status of each child at the time of enrollment was assessed and managed according to the WHO guidelines (9). Children in the intervention group (IG) received the oral probiotic in addition to the standard management. The probiotic was given as a single sachet (Wecare Probiotics Co., Ltd., production no.: SC10632050900407) containing LRa05 strain 5 × 10^9^ CFU/sachet, and was taken each day for seven consecutive days starting on the first day of clinical treatment.

Children in the control group (CG) were only treated with the standard therapy as mentioned above plus the reference sachet (placebo) containing only maltodextrin. The probiotic and placebo had similar appearance, taste, and smell and were provided in identical sachets with identical labeling expect for the subject specific randomization number. The children’s parents and/or guardians, clinicians, laboratory personnel, data manager, and statistician remained blinded to group assignments until the end of data analysis.

### Data collection

Following enrollment, the study staff performed assessments, documented data on clinical record form (CRF), and collected laboratory samples in accordance with the protocol. The clinicians used the CRF to document the incidence of abdominal cramps, nausea, vomiting, fever, constipation, and low appetite in the children during treatment. The mean of daily Bristol fecal score was calculated by dividing the sum of the daily Bristol fecal score by the fecal frequency in a given day.

### Fecal biochemical assessment

Commercial enzyme-linked immunosorbent assay (ELISA) kits (Shanghai Enzyme-linked Biotechnology Co., Ltd.) were used to measure sIgA, calprotectin, human beta-defensin 2 (HBD-2), and cathelicidin (LL-37) in fecal samples collected from all children before and after the intervention.

### Fecal microbiome analysis

A total of 158 fecal samples from the children were collected for gut microbiome analyses, including 82 samples from 41 children in the IG before and after the intervention and 76 samples from 38 children in the CG. Genomic DNA from the samples was extracted using a QIAamp Fast DNA fecal Mini Kit (Qiagen, Valencia, California, USA) with the CTAB/SDS method. The bacterial 16S rRNA gene V3-V4 region was amplified using the TransGen AP221-02 Kit (TransGen, Beijing, China), and the library was sequenced on an Illumina NovaSeq platform to generate 250 bp paired-end reads. Alpha- (within sample) and beta- (between sample) diversity were calculated using QIIME (Version 1.9.1). Shannon, Simpson, Chao1, and ACE indices were used as indicators of the alpha diversity, while beta diversity was analyzed by principal coordinate analysis (PCoA) based on Bray-Curtis distance. Linear discriminant analysis effect size (LEfSe) was used to analyze differential enrichment of gut microbiome. To explore the functional profiles of the gut microbiome, Phylogenetic Investigation of Communities by Reconstruction of Unobserved States (PICRUSt) was performed based on 16S information (10).

### Outcome measures

The primary outcome measure was the duration of diarrhea. The last abnormal feces was defined as the one after which the child passed normal or no feces for the next 24 h. Secondary outcome measures include the number of loose feces per day and the Bristol fecal score throughout the diarrhea episode, adverse effects, fecal biochemical indices, and fecal gut microbiome.

### Efficacy judgment

The evaluation of efficacy followed the national pediatric diarrhea efficacy evaluation standards and was consistent with previous studies (8, 11). Specifically, efficacy was determined by the reduction of diarrhea frequency to <4 times/day and the resolution of clinical symptoms after 72 h of treatment.

### Statistical analysis

All efficacy analyses were performed using both the intention-to-treat (ITT) dataset and per protocol dataset (PPS). Statistical analysis was performed using SAS version 9.2 for Windows (SAS Institute Inc., Cary, NC, USA).

For normally distributed data, a t-test was used for comparison, while Wilcoxon rank sum test was used for data without a normal distribution. Countable data were compared using the *χ*^2^ test to assess the difference in treatment efficacy between the two groups. Repeated measures ANOVA was used to compare the frequency of feces and the mean daily Bristol fecal score between the two groups before and after the intervention. A *p* < 0.05 was considered statistically significant.

### Sample size

In a previous study on the treatment of rotavirus enteritis with three combined strains (11), the diarrhea duration of the CG and the IG was 143.9 ± 19.8 h and 121.4 ± 13.7 h, respectively (almost reduced by 24 h). With *β* = 0.8, *α* = 0.05 (bilateral), the sample size of each group was calculated to be 50 subjects. Accounting for a 20% dropout rate, we selected a sample size of 120 subjects with 60 subjects in each group.

## Results

### Basic clinical and demographic data

**Figure 1:**
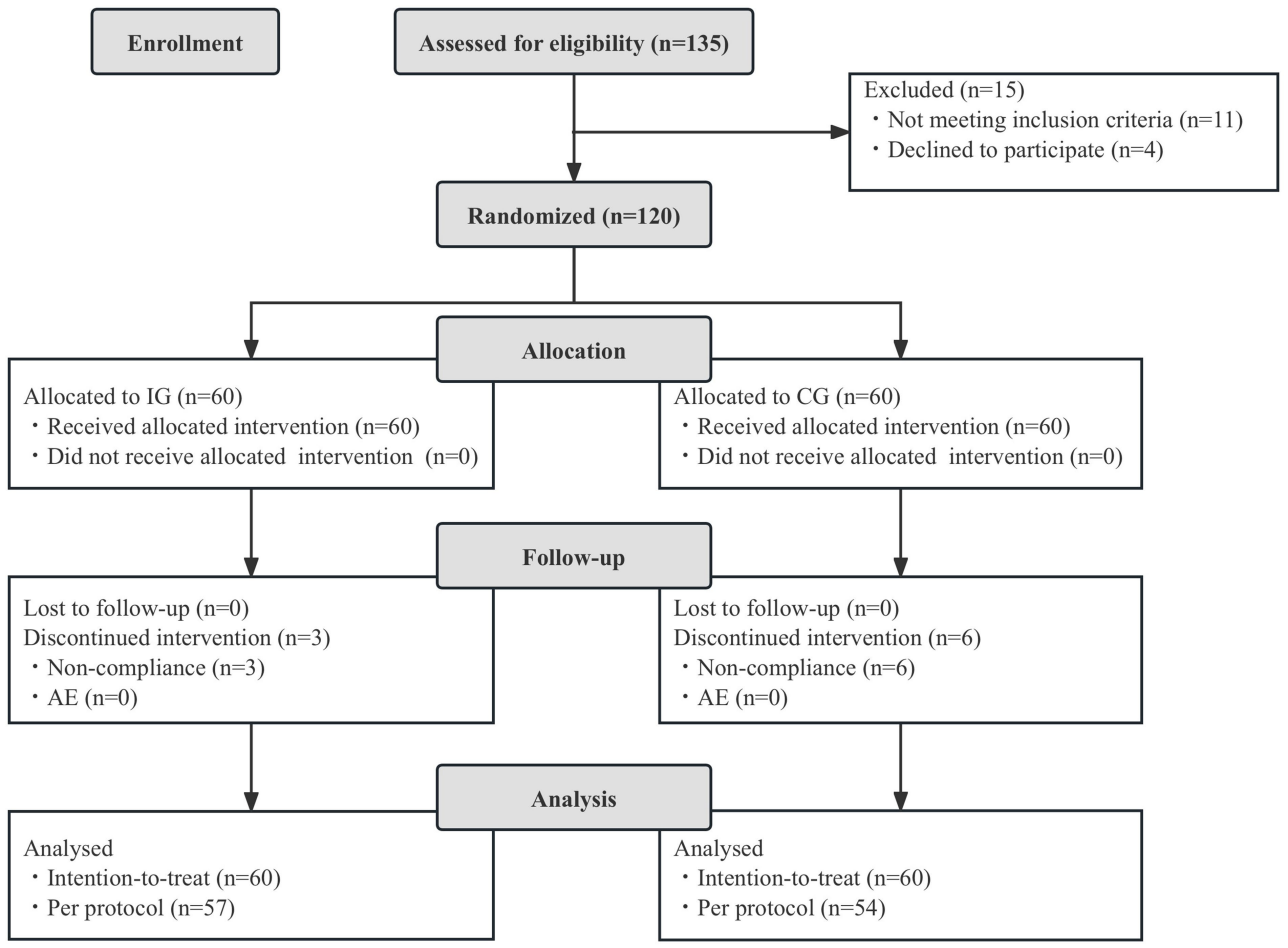
Flowchart of subject enrollment and study progress. IG, intervention group; CG, control group; AE, adverse events.

A total of 120 children were enrolled and randomized into the study and included in the ITT analysis. Sixty were randomized to the IG and 60 to the CG. No children were lost to follow up, and all children completed CRF. Ten children were excluded from the PP analysis due to major protocol deviations. The total of 110 infants were included in the PP dataset (57 in the IG and 54 in the CG). No adverse events related to study product intake were reported during the study. Figure 1 is a flowchart illustrating participant involvement. There was no significant difference in demographics, total and mean Bristol fecal score, and daily fecal frequency before intervention between the two groups (*p* > 0.05, Table 1).

**Table 1:** Basic clinical and demographic data between two groups before intervention [mean ± standard deviation or median (P25, p75)].

Items		IG	CG	*χ^2^* values	*p-*values
No.		57	54	–	–
Sex composition [male, *n* (%)]^*^		29 (50.9)	25 (46.3)	0.233	0.629
Age (m)^*^	Mean ± SD	10.97 ± 17.0	10.53 ± 16.2	0.140	0.445^+^
	Median (P25, P75)	1.1 (0.47, 15.33)	1.23 (0.47, 15.97)		
Full term or not [yes, *n* (%)]^*^		53 (93.0)	47 (87.0)	–	0.319^#^
Delivery mode [vaginal, *n* (%)]^*^		38 (66.7)	31 (57.4)	1.011	0.315
Slight dehydration [yes, *n* (%)]		3 (5.3)	1 (1.9)	–	0.396^#^
Registered residence [urban, *n* (%)]^*^		50 (87.7)	47 (87.0)	–	0.777^#^
Family history of allergic disease [yes, *n* (%)]		0	0	–	–
Previous allergic disease [yes, *n* (%)]		0	0	–	–

*There was no significant difference between the IG and the CG (*p* > 0.05); #, Fisher exact probability method; +, Wilcoxon non-parametric test between groups; IG, intervention group; CG, control group; SD, standard deviation.

### Efficiency of probiotic intervention on diarrhea

After the intervention, the total duration of diarrhea of children in the IG were significantly shorter than that of children in the CG [(122.4 ± 13.5) vs. (136.1 ± 14.2) hours, respectively, *p* < 0.001] (Table 2). The effective rate after 72 h of treatment was also significantly higher in the IG compared to the CG [70.2% (40/57) vs. 46.3% (25/54), *p* = 0.01] for PP analyses. These results were consistent for both ITT and PP analyses. The ITT analysis also showed that the effective rate in the IG was significantly higher than that in the CG [66.7% (40/60) vs. 41.7% (25/60), respectively, *χ*^2^ = 8.571, *p* = 0.003].

**Table 2:** Efficiency of probiotic intervention on diarrhea [*n* (%)].

Items		IG (*n* = 57)	CG (*n* = 54)	*χ^2^* values	*p*-values
No. of marked efficiency [*n* (%)]^*^		29 (50.9)	13 (24.1)	9.915	0.01
No. of normal efficiency [*n* (%)]^*^		11 (19.3)	12 (22.2)		
No. of inefficiency [*n* (%)]^*^		17 (29.8)	29 (53.7)		
No. of total efficiency [*n* (%)]^*^		40 (70.2)	25 (46.3)	6.516	0.01
Total duration of diarrhea (hours)^*^	Mean ± SD	122.4 ± 13.5	136.1 ± 14.2	5.204	<0.001
	Median (P25, P75)	120 (48, 144)	140 (48, 164)		

*Compared with the CG, the difference was statistically significant (*p* < 0.05); IG, intervention group; CG, control group; SD, standard deviation.

### Efficiency of probiotic intervention on daily fecal frequency

**Figure 2:**
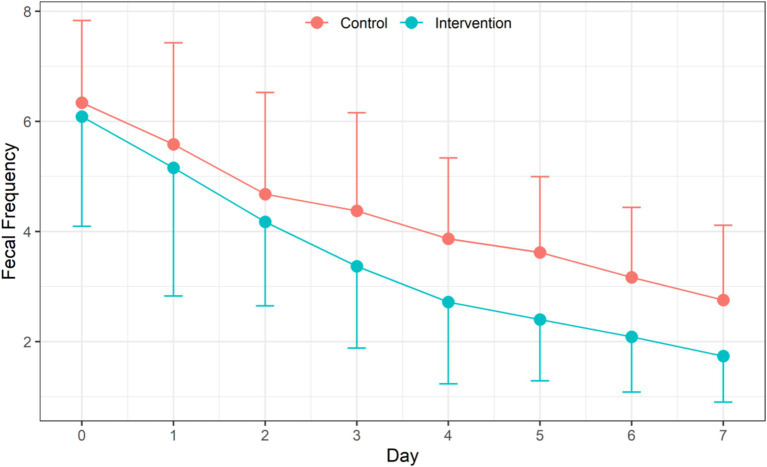
Efficiency of probiotic intervention on daily fecal frequency of children between two groups.

Daily frequency of feces within each group decreased significantly (*F* = 201.39, *p* < 0.001) with the extension of treatment time. There was also a significant difference in the daily frequency of feces between the IG and the CG. The frequency of feces in the IG was significantly less than that in the CG (*F* = 11.89, *p* = 0.0005). There was also a significant interaction between treatment time and intervention method (*F* = 3.77, *p* = 0.0005) (Table 3 and Figure 2).

**Table 3:** Efficiency of probiotic intervention on daily fecal frequency of children between two groups [mean ± standard deviation, median (P25, P75)].

Daily fecal frequency			IG (*n* = 57)	CG (*n* = 54)
One day before intervention		Mean ± SD	6.09 ± 1.99	6.33 ± 1.50
		Median (P25, P75)	6 (5, 8)	6 (5, 7)
1st day during intervention		Mean ± SD	5.16 ± 2.33	5.57 ± 1.83
		Median (P25, P75)	5 (4, 7)	6 (4, 6)
2nd day during intervention		Mean ± SD	4.18 ± 1.53	4.69 ± 1.86
		Median (P25, P75)	4 (3, 5)	5 (4, 6)
3rd day during intervention		Mean ± SD	3.37 ± 1.48	4.39 ± 1.77
		Median (P25, P75)	3 (2, 4)	4 (3, 6)
4th day during intervention		Mean ± SD	2.72 ± 1.49	3.88 ± 1.46
		Median (P25, P75)	2 (2, 4)	4 (3, 5)
5th day during intervention		Mean ± SD	2.40 ± 1.12	3.62 ± 1.39
		Median (P25, P75)	2 (2, 3)	4 (3, 5)
6th day during intervention		Mean ± SD	2.09 ± 1.01	3.19 ± 1.30
		Median (P25, P75)	2 (1, 3)	3 (2, 4)
7th day during intervention		Mean ± SD	1.74 ± 0.84	2.79 ± 1.37
		Median (P25, P75)	2 (1, 2)	3 (2, 4)
Time efficiency	*F*-value^*^	201.39		
	*p*-value	<0.001		
Intervention efficiency	*F*-value^*^	11.89		
	*p*-value	0.0005		
Time-intervention interaction efficiency	*F*-value^*^	3.77		
	*p*-value	0.0005^**^		

*Analysis of variance of repeated measurement data; IG, intervention group; CG, control group; SD, standard deviation; **Post-hoc analysis.

### Efficiency of probiotic intervention on the mean of daily Bristol fecal score of children

**Figure 3:**
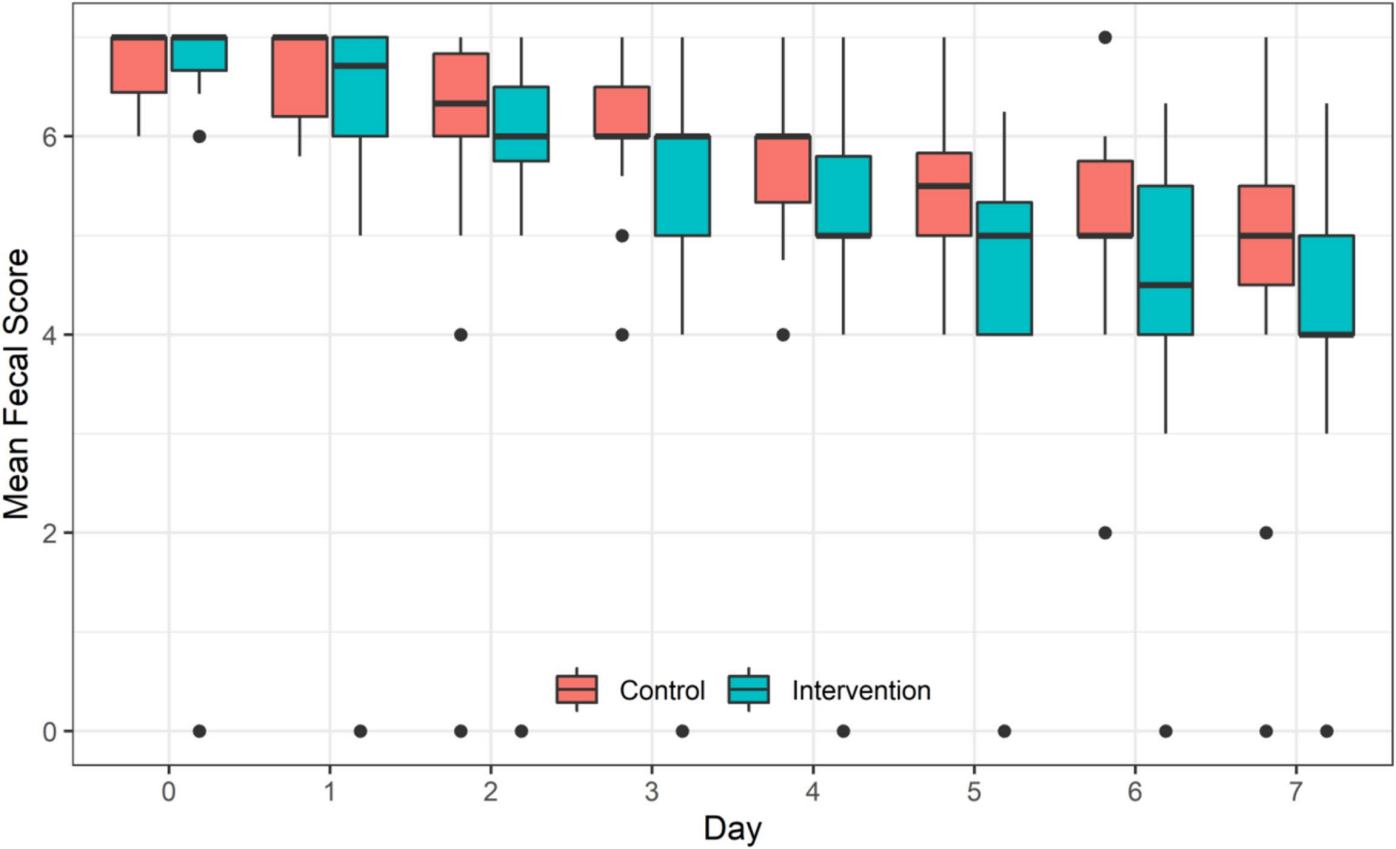
Efficiency of probiotic intervention on the mean of daily Bristol fecal score of children between the two groups.

The repeated measures ANOVA showed that with the extension of treatment time, the mean of daily Bristol fecal score of children in both groups decreased significantly (*F* = 185.66, *p* < 0.001). Children in the IG had significantly lower mean of daily Bristol fecal score than children in the CG (*F* = 18.93, *p* = 0.0061). A significant interaction between the intervention and treatment time was observed (*F* = 3.09, *p* = 0.0033) (Table 4 and Figure 3).

**Table 4:** Efficiency of probiotic intervention on the mean of daily Bristol fecal score of children between the two groups [mean ± standard deviation, median (P25, P75)].

Mean of daily Bristol fecal score			IG (*n* = 57)	CG (*n* = 54)
One day before intervention		Mean ± SD	6.64 ± 0.97	6.71 ± 0.54
		Median (P25, P75)	7 (6.67, 7)	7 (6.3, 7)
1st day during intervention		Mean ± SD	6.31 ± 1.32	6.76 ± 0.51
		Median (P25, P75)	6.71 (6, 7)	7 (6.6, 7)
2nd day during intervention		Mean ± SD	5.92 ± 0.98	6.22 ± 1.09
		Median (P25, P75)	6 (5.75, 6.5)	6.33 (6, 6.73)
3rd day during intervention		Mean ± SD	5.60 ± 0.99	6.06 ± 0.67
		Median (P25, P75)	6 (5,6)	6 (6, 6.7)
4th day during intervention		Mean ± SD	4.96 ± 1.41	5.70 ± 0.74
		Median (P25, P75)	5 (5, 5.8)	6 (5.2, 6)
5th day during intervention		Mean ± SD	4.82 ± 1.00	5.38 ± 0.67
		Median (P25, P75)	5 (4, 5.33)	5.5 (5.0, 5.67)
6th day during intervention		Mean ± SD	4.67 ± 1.04	5.15 ± 0.78
		Median (P25, P75)	4.5 (4.0, 5.5)	5 (5, 5.78)
7th day during intervention		Mean ± SD	4.64 ± 0.85	5.02 ± 0.77
		Median (P25, P75)	4.25 (4.0, 5.0)	5 (5, 5.5)
Time efficiency	*F*-value^*^	185.66		
	*p*-value	<0.001		
Intervention efficiency	*F-*value^*^	18.93		
	*p*-value	0.0061		
Time-intervention interaction efficiency	*F*-value^*^	3.09		
	*p*-value	0.0033^**^		

*Analysis of variance of repeated measurement data; IG, intervention group; CG, control group; SD, standard deviation; **Post-hoc analysis.

### Efficiency of probiotic intervention on fecal biochemical indices of children

**Figure 4:**
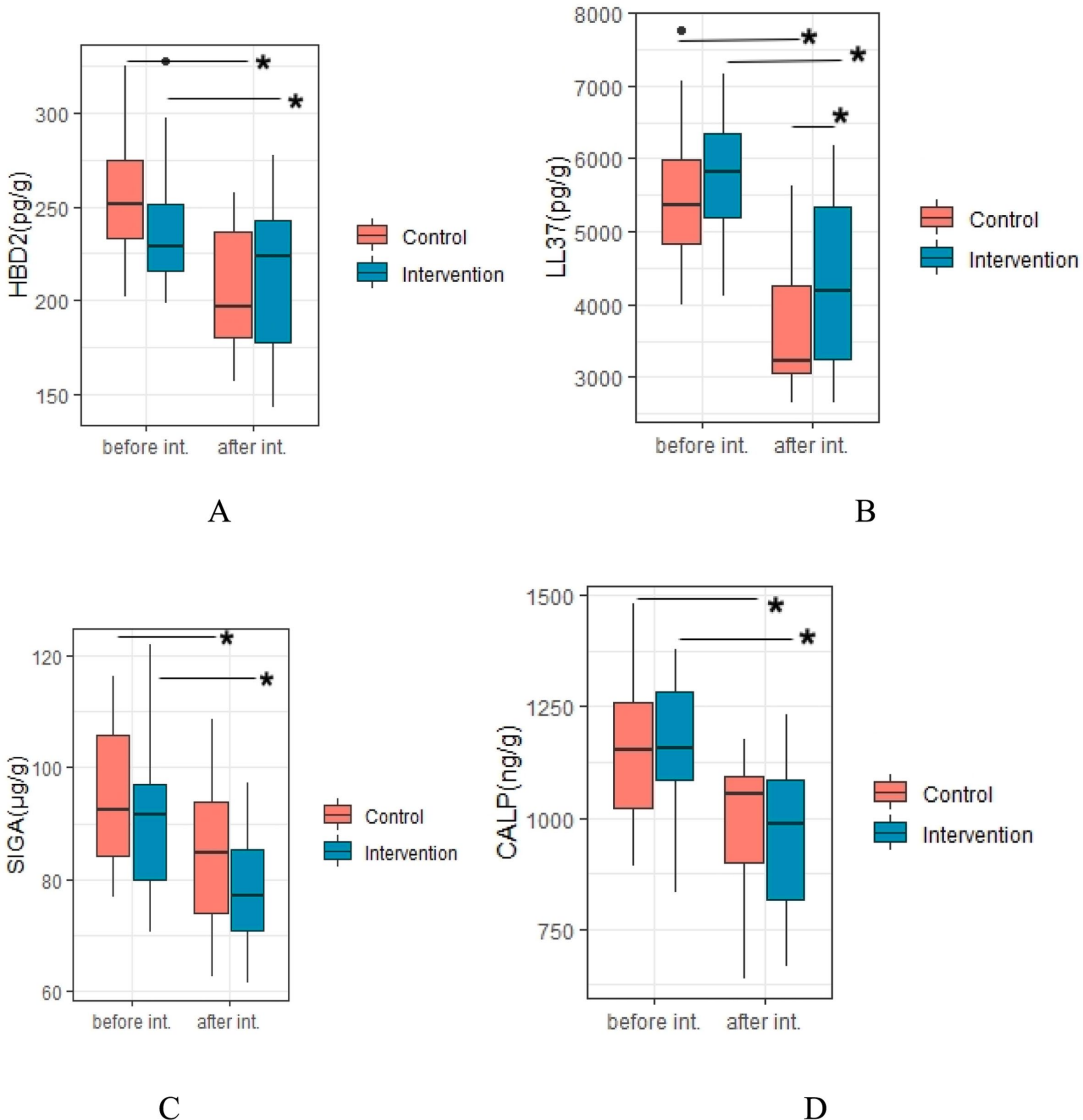
Efficiency of probiotic intervention on fecal biochemical indices of children. *Difference with statistical significance; (A), HBD-2, human beta-defensin 2; (B), LL37, cathelicidin (LL-37); (C), SIgA, sIgA; (D), CALP, calprotectin; int. Intervention; Intervention, IG group; Control, CG group.

A total of 22 and 19 of children in IG and CG groups, respectively who collected enough fecal samples before and after the intervention to measure biochemical indicators slgA, calprotectin, HBD-2 and LL-37 levels (Figure 4). After intervention, levels of these fecal biochemical indices were all significantly decreased when compared to the baseline level (all *p* < 0.05) in both groups, while level of LL-37 of children in IG was markedly higher than that of children in CG (4349.35 ± 1143.86 pg./g vs. 3682.49 ± 869.21 pg./g, *p* = 0.039). Nevertheless, the differences of slgA, calprotectin, and HBD-2 levels after intervention between the two groups was not significant (all *p* > 0.05).

### Efficiency of probiotic intervention on fecal gut microbiota

**Figure 5:**
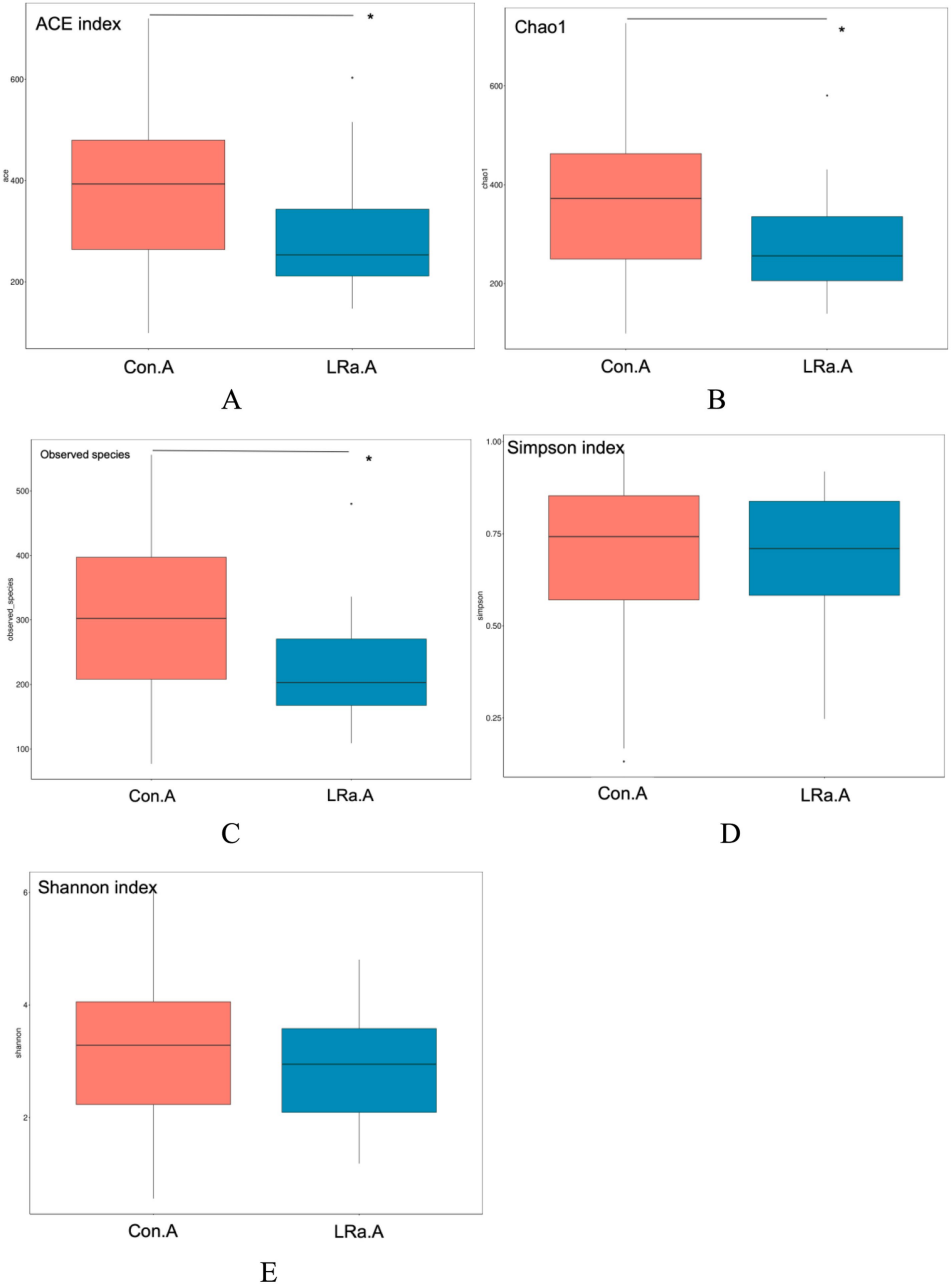
Efficiency of probiotic intervention on alpha diversity indices of the gut microbiota between the two groups after the intervention. *Significant difference between the two groups; (A), ACE index; (B), chao1; (C), observed species; (D), Simpson index; (E), Shannon index; Con.A, CG after intervention; LRa.A, IG after intervention.

As shown in Figure 5, analysis of alpha diversity revealed that the richness estimates (calculated in observed species, ACE and Chao1 indices) in the IG were significantly lower than those in the CG after the intervention (all *p* < 0.01), however no significant difference in Shannon and Simpson indices was found between the groups after the intervention (*p* = 0.381 and 0.685, respectively).

**Figure 6:**
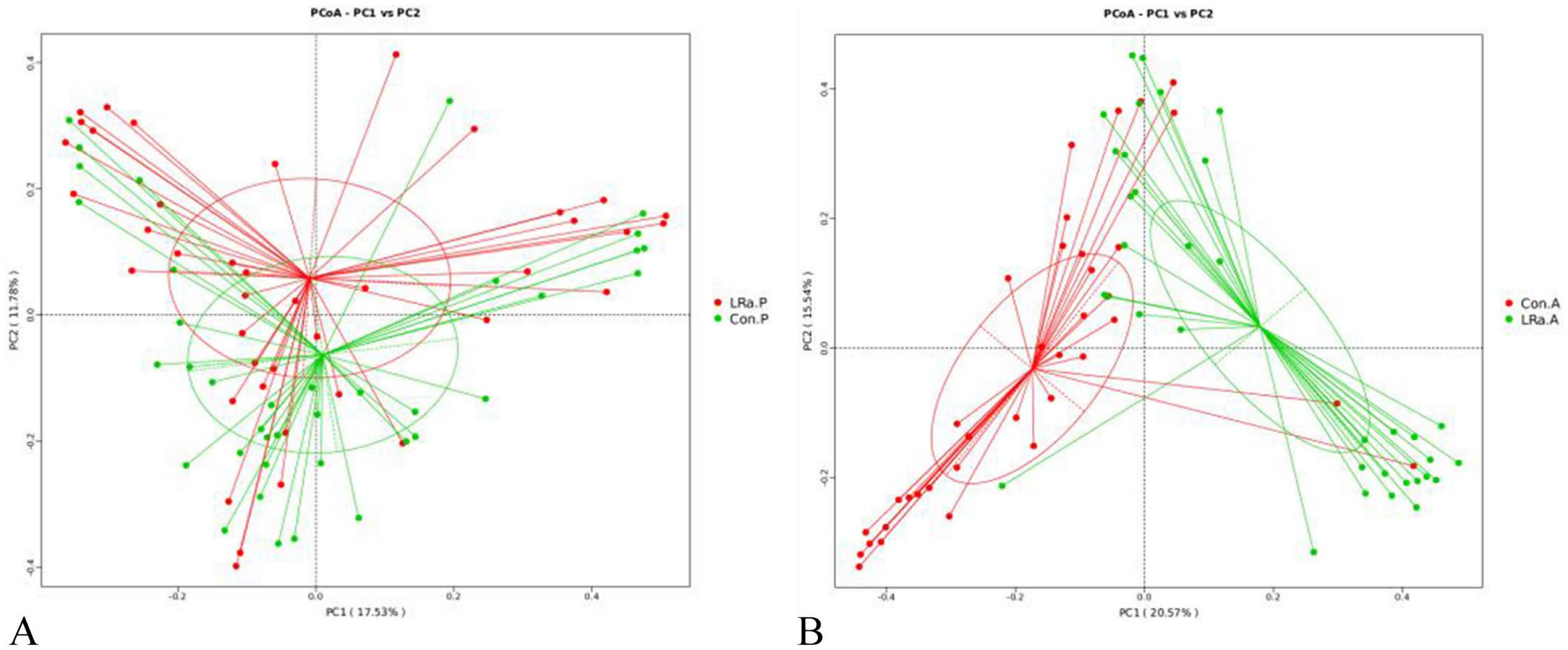
Analysis of the beta diversity calculated on the Principal coordinate analysis (PCoA) based on Bray-Curtis distance (A), before intervention; (B), after intervention; Con.P, control group before intervention; LRa.P, intervention group before intervention; Con.A, control group after intervention; LRa.A, intervention group after intervention.

The PCoA plot based on Bray-Curtis distance showed that axis 1 (PC1) explained 17.53% of the variability and axis 2 (PC2) explained 11.78% of the variability of before intervention. The PCoA plot demonstrated that the samples of children in IG and CG were spatially close to each other (Figure 6A). While, after intervention, the samples from the two groups were spatially separated (Figure 6B).

**Figure 7:**
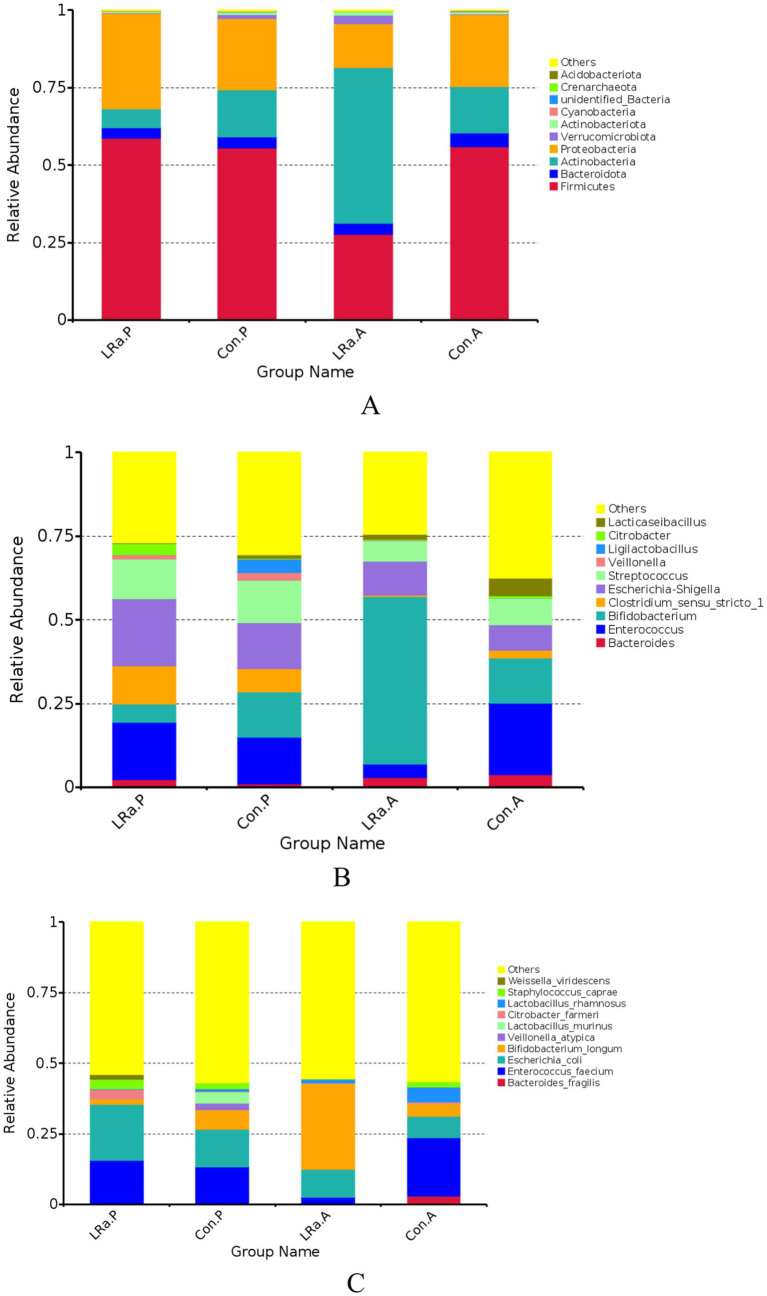

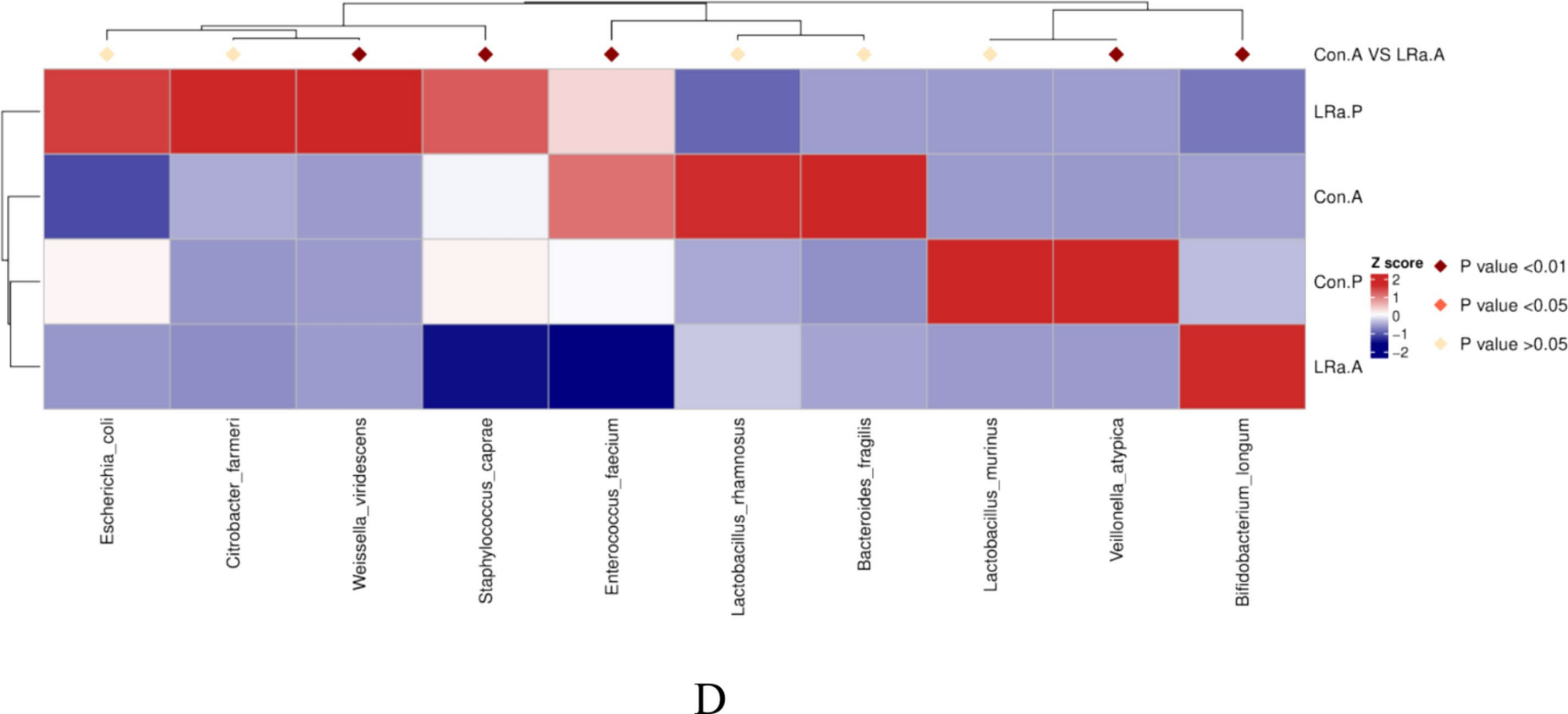
Taxa abundance at phylum (A), genus (B) and species (C) levels. Only the top 10 most abundant phyla, genera, and species were shown. (D) The MetaStat Complex Heat map showing the differential abundance between the two groups with statistical significance. Con.A, control group after intervention; LRa.A, intervention group after intervention; Con.P, control group before intervention; LRa.P, intervention group before intervention.

The gut microbiome composition was presented in Figure 7. After the intervention, the dominate phylum, genus, and species changed to Actinobacteria, *Bifidobacterium*, and *Bifidobacterium longum*, respectively, in IG group. Furthermore, the MetaStat method confirmed that the abundance of *Bifidobacterium longum*, *Veillonella_atypicain* and *Weissella_viridescens* in the IG was significantly higher than that in the CG (*p* < 0.05) (Figure 7).

**Figure 8:**
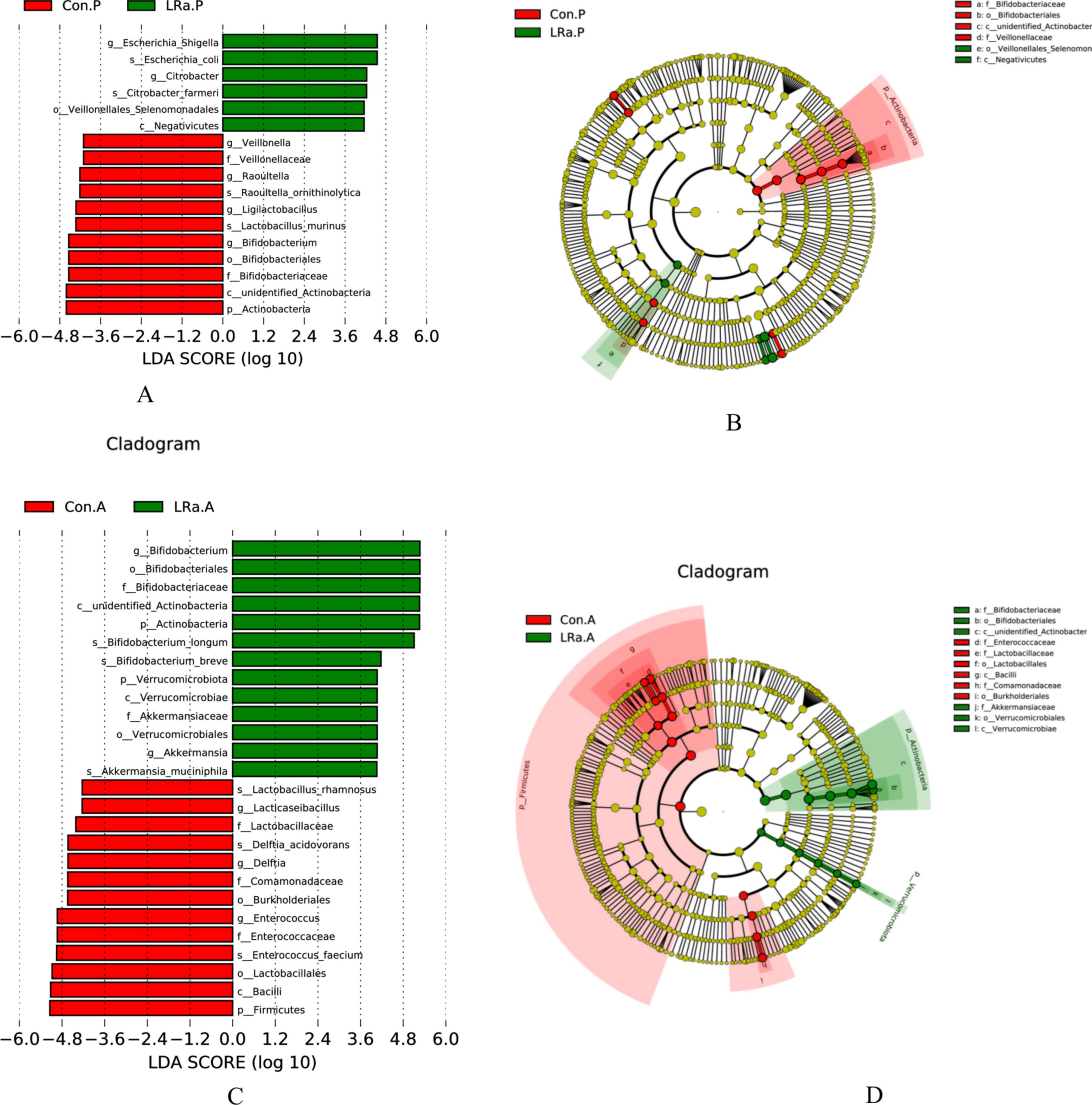
LEfSe analysis identified the most deferentially abundant taxa between the intervention and control groups. Cladogram: Taxonomic representation of statistically and biologically consistent differences among intestinal microbiota of different groups. Differences were represented by the color of the most abundant taxa (Green indicated a taxon with significantly higher relative abundance in the intervention group, red indicated a taxon significantly more abundant in the control group and yellow indicated no significant difference). LAD SCORE: Histogram of linear discriminant analysis (LDA) scores for deferentially abundant taxon. Cladogram was calculated by LefSe and displayed according to effect size. (A,B) LDA score and Cladogram before intervention; (C,D) LDA score and Cladogram after intervention; Con.A, control group after intervention; LRa.A, intervention group after intervention; Con.P, control group before intervention; LRa.P, intervention group before intervention.

LEfSe analysis identified only 6 taxa that were differentially abundant between the two groups before the intervention. However, there were 12 differentially abundant taxa after the intervention (Figure 8). In comparison to the CG, the LRa05 treatment increased the abundance of 2 families (Bifidobacteriaceae and Akkermansiaceae), 2 orders (Bifidobacteriales and Verrucomicrobiales), and 2 classes (unidentified Actinobacter, and Verrucomicrobiae). Furthermore, LDA scores (>4.0) identified notable high abundance in the *Bifidobacterium*, *Akkermansia*, and *Ruminococcus* genera and *Bifidobacterium longum*, *Bifidobacterium breve*, and *Akkermansia muciniphila* species in children from the IG.

### LRa05 treatment changed the functional gene composition of gut microbiota

**Figure 9:**
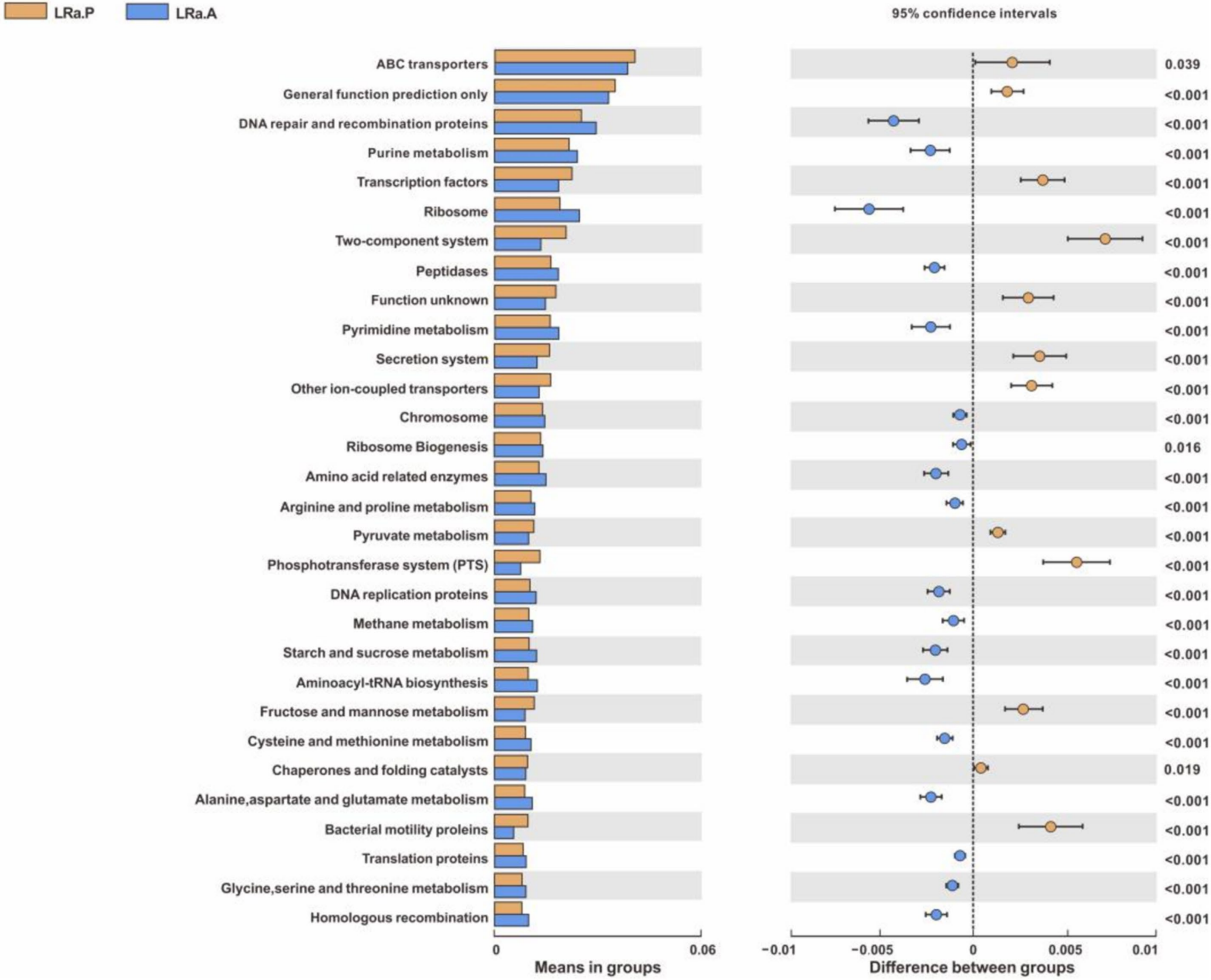
PICRUSt function prediction of the gut microbiota in the LRa05 group with top 30 means in groups (Welch’s t test, two-sided, *p* < 0.05) LRa.A, intervention group after intervention; LRa.P, intervention group before intervention.

PICRUSt result showed the proportion of 108 sub-functional genes of gut microbiome was evidently changed after LRa05 treatment only with top 30 means in groups shown, such as DNA repair and recombination protein, purine metabolism, ribosome, peptidases, pyrimidine metabolism, chromosome, ribosome biogenesis and amino acid related enzymes (Figure 9).

## Discussion

The LRa05 strain has been applied for patents to relieve diarrhea (12) and constipation, mediate intestinal flora (13), enhance immunity, or improve eczema (14). Studies have shown that LRa05 can reshape the intestinal microflora by increasing the number of bacteria producing short chain fatty acids (SCFA) and reducing the number of proinflammatory bacteria (15).

### Efficiency of probiotic intervention on diarrhea, fecal consistency, and frequency

Reducing the duration of diarrhea and hospital stay are important aims in the treatment of acute diarrhea from medical, social, and economical perspectives (7). However, fluid and electrolyte replenishment could not substantially reduce the frequency and/or shorten the duration of diarrhea (16). Several studies have reported that microorganisms, such as *L. rhamnosus* GG, *Saccharomyces boulardii*, *Lactobacillus reuteri*, and *Bifidobacterium lactis* Bi-07 combined with *Lactobacillus rhamnosus* HN001, *Lactobacillus acidophilus* NCFM, *Saccharomyces boulardii* CNCM I-745 and CNCM I-3799, *Lactobacillus reuteri* NCIMB 30351, and *Lactobacillus plantarum* LRCC5310 are effective in treating acute diarrhea (11, 17–24).

It is worth noting that the good adjuvant effect of LRa05 in the treatment for acute watery diarrhea had manifested on the second day of the treatment, which is consistent with the results of a similar probiotic intervention (25). In detail, After 3 days treatment, the daily fecal frequency of children in the IG was 1.01 times less than that of children in the CG. The efficiency was most significant on the fifth day with an average reduction of 1.23 times in fecal frequency. The reduced fecal frequency may be attributed to three factors: the time effect, the intervention effect, and the interaction between the two. Firstly, the daily fecal frequency in both groups had decreased significantly with the extension of treatment time, which indicated that the treatment in both groups were effective. Secondly, the probiotic intervention exhibited additional beneficial effects of relieving the diarrhea after excluding the time effect. Finally, the interaction effect showed that with the extension of treatment time, the reduction of fecal frequency in the probiotic intervention group was more efficient. Besides the reduced frequency of diarrhea, the probiotic significantly improved the consistency of feces. The changes in fecal consistency were very similar to the changes in fecal frequency showing an intra-group effect of intervention time, an inter-group effect of probiotic intervention, and an interaction between the time and probiotic treatment.

Moreover, during the 7-day intervention, the children in neither the IG nor the CG had abdominal colic, nausea, vomiting, fever, and low appetite related to the use of probiotics, indicating the safety of LRa05 for infants with diarrhea.

### Efficiency of probiotic intervention on fecal biochemical indices

The changes of these immunity biomarkers in this study are different from other studies using different strains, but it is very similar to the results of our previous interventions using the other two specific strains, *Bifidobacterium animalis* sp. Lactis XLTG11 (26) and *Bifidobacterium animalis* subsp. lactis BLa80 for acute diarrhea (27). For example, Nocerino et al. (28) and one of our previous studies (29) have shown that *Bifidobacterium animalis* BB-12 can significantly increase these fecal immunity biomarkers, suggesting that this strain can improve the intestinal immune characteristics.

The decreased HBD-2, LL-37, and sIgA levels after the intervention may be due to the following reasons. Some studies (30–32) have shown that the immunity biomarkers are significantly increased to fight against the dominant or potential inflammation of the intestine. This is confirmed by our results that no difference in these biomarkers was observed before the intervention. Their intestinal symptoms were rapidly improved and therefore the decline in immunity biomarkers was expected. Secondly, the acute diarrhea for infants has a characteristic of rapid recovery and the interval between the two collections of feces was only 7 days. After the diarrhea was controlled, there might be no enough time for the immunity biomarkers to recover to the pre-disease state, let alone the immunomodulation effect of the probiotic. Therefore, the regulatory effect of the probiotic on intestinal immunity may not be manifested. If the observation time is extended, different changes of these immunity biomarkers after intervention between the two groups might be observed. Thirdly, this study was a clinical trial. The initial sample size was calculated based on clinical indicators, such as fecal frequency. The sample size of fecal analysis might be too small to distinguish the significant differences between the two groups after the intervention. In addition, the change in calprotectin (33, 34), which reflects the inflammatory state of the intestine, can be explained similarly as the immunity biomarkers.

### Efficiency of probiotic intervention on gut microbiota

The gut microbiota composition change of intestinal microecology in children with diarrhea and the influence of probiotics on intestinal microecology are closely related to the therapeutic effect and clinical process of diarrhea. Recent studies on the use of *Lactobacillus rhamnosus*, as an adjuvant therapy for gastrointestinal diseases in children, have shown that the use of *Lactobacillus rhamnosus* can regulate the intestinal microecological composition and improve the prognosis (33, 35, 36). However, the effecst of different specific strains on the composition of intestinal flora were very different. For example, *Lactobacillus casei* variety *rhamnosus* was beneficial for the counts of *Bifidobacteria* and *Lactobacillus* species, while long-term *L. rhamnosus* GG supplementation can cause an increase in the abundance of *Prevotella*, *Lactococcus*, and *Ruminococcus*, and a decrease in *Escherichia*, *lactobacilli/enterococci* and *clostridia* in feces. Our previous studies domesticated that after the intervention of *Bifidobacterium animalis* sp. Lactis XLTG11 for 1 week, Actinobacteria, *Bifidobacterium*, and *B. longum* became the dominate phylum, genus, and species, respectively, in the children of intervention group (26), while Proteobacteria, *Enterococcus*, and *Enterococcus faedum* remained dominant in the children of control group while for the dominate *Bifidobacterium breve* and *Collinsella aerofaciens* species for Bla80 intervention (27). Similar to the above results, the present study also showed that the LRa05 administration can change the gut microbiome composition into a gut microbiome dominated by the phylum Actinobacteria, genus *Bifidobacterium*, and species *Bifidobacterium longum* and with low species abundance of *Enterococcus faecium*, *Staphylococcus caprae*, and *Delftia acidovorans*. The above changes in intestinal microecology composition were accompanied by the improvement of clinical manifestation in children with diarrhea, which suggested that the use of LRa05 has beneficial impact on intestinal microecology of children with diarrhea.

Although the diarrhea of the infants in the CG also recovered after the treatment, their gut microbiome composition was completely different from that of the infants in the IG. Not only did the diversity of gut microbiome increase, but also the abundance of some potential pathogens increased. Whether the different gut microbiome composition between the two groups will affect the incidence rate of diarrhea in a later period warrants a long-term follow-up study.

According to functional gene prediction analysis, LRa05 treatment upregulated the functional genes involved in the purine metabolism of gut microbiome, and the extracellular purines play a pivotal role (37) in controlling the chemotaxis, activation, proliferation, and differentiation of immune cells. Some of the studies (38–40) has established a connection between nucleolar activity, ribosome abundance, and cell intrinsic immunity which proved that ribosome biogenesis unexpectedly can regulate dsDNA-sensing to restrict virus reproduction and regulate inflammation. Totally, these results indicated that LRa05 treatment might regulate the immunity related genes of the gut microbiome and might contribute to mitigating the symptoms of patients with diarrhea.

### Limitation analysis

Firstly, the present study did not detect the possible viral and bacterial pathogens that caused the watery diarrhea, so it cannot further explore the different responses of specific pathogens to the LRa05 administration. Secondly, using a single dose of LRa05 at 5 × 10^9^ CFU/day prevented us from exploring the optimal dose–response relationship of LRa05 strain in the adjuvant treatment of watery diarrhea. Thirdly, due to the limitations of the main objectives and design of the study, the duration of the probiotic intervention and clinical symptom observation was only 1 week, so the possible long-term effects of LRa05 on children’s health and gut microbiome cannot be observed.

## Conclusion

To conclude, we did not observe any adverse effects of LRa05 intervention during our study period, which indicated the safety of LRa05 for infants. Administration of the *Lacticaseibacillus rhamnosus* LRa05 at a dose of 5 × 10^9^ CFU/day to children aged 0–3 years resulted in shorter duration of diarrhea, faster improvement in fecal consistency, and beneficial changes in gut microbiome composition and gene functions.

## Data Availability

Raw 16S rRNA gene sequence for all feces samples used in this study have been deposited in the National Center for Biotechnology Information BioProject database with the BioProject ID PRJNA1184113 (https://dataview.ncbi.nlm.nih.gov/object/PRJNA1184113?reviewer=l3hmkrciq0nda2k9a6sau4hoo3).

## References

[1] Riddle MS Martin GJ Murray CK Burgess TH Connor P Mancuso JD. Management of Acute Diarrheal Illness during Deployment: a deployment health guideline and expert panel report. Mil Med. (2017) 182:34–52. doi: 10.7205/MILMED-D-17-00077, PMID: 28885922 PMC5657341

[2] Chapman CMC Gibson GR Rowland I. Health benefits of probiotics: are mixtures more effective than single strains? Eur J Nutr. (2011) 50:1–17. doi: 10.1007/s00394-010-0166-z, PMID: 21229254

[3] Collado MC Isolauri E Salminen S. Specific probiotic strains and their combinations counteract adhesion of *Enterobacter sakazakii* to intestinal mucus. FEMS Microbiol Lett. (2008) 285:58–64. doi: 10.1111/j.1574-6968.2008.01211.x, PMID: 18503543

[4] Drago L De Vecchi E Gabrieli A De Grandi R Toscano M. Immunomodulatory effects of *Lactobacillus salivarius* LS01 and *Bifidobacterium breve* BR03, alone and in combination, on peripheral blood mononuclear cells of allergic asthmatics. Allergy Asthma Immunol Res. (2015) 7:409–13. doi: 10.4168/aair.2015.7.4.409, PMID: 25749784 PMC4446640

[5] Drago L Gismondo MR Lombardi A de Haën C Gozzini L. Inhibition of in vitro growth of enteropathogens by new Lactobacillus isolates of human intestinal origin. FEMS Microbiol Lett. (1997) 153:455–63. doi: 10.1111/j.1574-6968.1997.tb12610.x, PMID: 9271875

[6] McFarland LV Evans CT Goldstein EJC. Strain-specificity and disease-specificity of probiotic efficacy: a systematic review and Meta-analysis. Front Med. (2018) 5:124. doi: 10.3389/fmed.2018.00124, PMID: 29868585 PMC5949321

[7] Guarino A Ashkenazi S Gendrel D Lo Vecchio A Shamir R Szajewska H. European Society for Pediatric Gastroenterology, hepatology, and nutrition/European Society for Pediatric Infectious Diseases evidence-based guidelines for the management of acute gastroenteritis in children in Europe: update 2014. J Pediatr Gastroenterol Nutr. (2014) 59:132–52. doi: 10.1097/MPG.0000000000000375, PMID: 24739189

[8] Hesong Fang EA. Summary of "98 National Symposium on diarrhea prevention and treatment", supplementary suggestions on new principles of diarrhea treatment and efficacy judgment criteria. J Clin Pediatr. (1998) 5:358

[9] Falszewska A Dziechciarz P Szajewska H. Diagnostic accuracy of clinical dehydration scales in children. Eur J Pediatr. (2017) 176:1021–6. doi: 10.1007/s00431-017-2942-8, PMID: 28573405

[10] Langille MGI Zaneveld J Caporaso JG McDonald D Knights D Reyes JA. Predictive functional profiling of microbial communities using 16S rRNA marker gene sequences. Nat Biotechnol. (2013) 31:814–21. doi: 10.1038/nbt.2676, PMID: 23975157 PMC3819121

[11] Chen K Xin J Zhang G Xie H Luo L Yuan S. A combination of three probiotic strains for treatment of acute diarrhoea in hospitalised children: an open label, randomised controlled trial. Benef Microbes. (2020) 11:339–46. doi: 10.3920/BM2020.0046, PMID: 32720832

[12] Wu T Zhang Y Li W Zhao Y Long H Muhindo EM. *Lactobacillus rhamnosus*lra05 ameliorate hyperglycemia through a regulating glucagon-mediated signaling pathway and gut microbiota in type 2 diabetic mice. J Agric Food Chem. (2021) 69:8797–806. doi: 10.1021/acs.jafc.1c02925, PMID: 34340304

[13] Shuguang F. Application of *Lactobacillus rhamnosus* lra05 in the preparation of products for relieving constipation or regulating intestinal flora. Chin Patent. (2022) 31:2022103825693

[14] Shuguang F. Application of *Lactobacillus rhamnosus* strain lra05 in the preparation of products to enhance immunity and / or alleviate eczema. Chin Patent. (2022):202210382762

[15] Sun M Wu T Zhang G Liu R Sui W Zhang M. LRa05 improves lipid accumulation in mice fed with a high fat diet regulating the intestinal microbiota, reducing glucose content and promoting liver carbohydrate metabolism. Food Funct. (2020) 11:9514–25. doi: 10.1039/D0FO01720E, PMID: 33063800

[16] Vandenplas Y Salvatore S Viera M Devreker T Hauser B Hauser B. Probiotics in infectious diarrhoea in children: are they indicated? Eur J Pediatr. (2007) 166:1211–8. doi: 10.1007/s00431-007-0497-9, PMID: 17611775

[17] Guarino A Guandalini S Lo Vecchio A. Probiotics for prevention and treatment of diarrhea. J Clin Gastroenterol. (2015) 49:S37–45. doi: 10.1097/MCG.0000000000000349 26447963

[18] Floch MH Walker WA Madsen K Sanders ME Macfarlane GT Flint HJ. Recommendations for probiotic use-2011 update. J Clin Gastroenterol. (2011) 45:S168–71. doi: 10.1097/MCG.0b013e318230928b, PMID: 21992958

[19] Guarner F Khan AG Garisch J Eliakim R Gangl A Thomson A. World gastroenterology organisation global guidelines. J Clin Gastroenterol. (2012) 46:468–81. doi: 10.1097/MCG.0b013e3182549092 22688142

[20] McFarland LV. Unraveling the causes of negative studies: a case of S boulardii for the prevention of antibiotic-associated diarrhea. Rev Med Chile. (2009) 137:719–20. doi: 10.4067/S0034-98872009000500021, PMID: 19701565

[21] Altcheh J Carosella MV Ceballos A D'Andrea U Jofre SM Marotta C. Randomized, direct comparison study of Saccharomyces boulardii CNCM I-745 versus multi-strained *Bacillus clausii* probiotics for the treatment of pediatric acute gastroenteritis. Medicine (Baltimore). (2022) 101:e30500. doi: 10.1097/MD.0000000000030500, PMID: 36086703 PMC9646502

[22] Mourey F Sureja V Kheni D Shah P Parikh D Upadhyay U. A multicenter, randomized, double-blind, placebo-controlled trial of Saccharomyces boulardii in infants and children with acute diarrhea. Pediatr Infect Dis J. (2020) 39:e347–51. doi: 10.1097/INF.0000000000002849, PMID: 32796401 PMC7556239

[23] Tyrsin OY Tyrsin DY Nemenov DG Ruzov AS Odintsova VE Koshechkin SI. Effect of *Lactobacillus reuteri* NCIMB 30351 drops on symptoms of infantile functional gastrointestinal disorders and gut microbiota in early infants: results from a randomized, placebo-controlled clinical trial. Eur J Pediatr. (2024) 183:2311–24. doi: 10.1007/s00431-024-05473-y, PMID: 38427038

[24] Shin DY Yi DY Jo S Lee YM Kim J-H Kim W. Effect of a new *Lactobacillus plantarum* product, LRCC5310, on clinical symptoms and virus reduction in children with rotaviral enteritis. Medicine (Baltimore). (2020) 99:e22192. doi: 10.1097/MD.0000000000022192, PMID: 32957348 PMC7505315

[25] Allen SJ Martinez EG Gregorio GV Dans LF Cochrane Infectious Diseases Group. Probiotics for treating acute infectious diarrhoea. Cochrane Database Syst Rev. (2010) 2010:CD003048. doi: 10.1002/14651858.CD003048.pub3, PMID: 21069673 PMC6532699

[26] Chen K Jin S Ma Y Cai L Xu P Nie Y. Adjunctive efficacy of Lactis XLTG11 for acute diarrhea in children: a randomized, blinded, placebo-controlled study. Nutrition. (2023) 111:112052. doi: 10.1016/j.nut.2023.112052, PMID: 37172455

[27] Chen K Jin S Ma Y Cai L Xu P Nie Y. Adjudicative efficacy of *Bifidobacterium animalis* subsp. lactis BLa80 in treating acute diarrhea in children: a randomized, double-blinded, placebo-controlled study. Eur J Clin Nutr. (2024) 78:501–8. doi: 10.1038/s41430-024-01428-6, PMID: 38467857 PMC11182741

[28] Nocerino R De Filippis F Cecere G Marino A Micillo M Di Scala C. The therapeutic efficacy of *Bifidobacterium animalis* subsp. lactis BB-12 in infant colic: a randomised, double blind, placebo-controlled trial. Aliment Pharmacol Ther. (2020) 51:110–20. doi: 10.1111/apt.15561, PMID: 31797399 PMC6973258

[29] Chen K Zhang G Xie H You L Li H Zhang Y. Efficacy of *Bifidobacterium animalis* subsp. lactis, BB-12(®) on infant colic - a randomised, double-blinded, placebo-controlled study. Benef Microbes. (2021) 12:531–40. doi: 10.3920/BM2020.0233, PMID: 34550055

[30] Huang F-C. Differential regulation of interleukin-8 and human beta-defensin 2 in *Pseudomonas aeruginosa*-infected intestinal epithelial cells. BMC Microbiol. (2014) 14:275. doi: 10.1186/s12866-014-0275-6, PMID: 25433669 PMC4261737

[31] Shirin T Rahman A Danielsson Å Uddin T Bhuyian TR Sheikh A. Antimicrobial peptides in the duodenum at the acute and convalescent stages in patients with diarrhea due to *Vibrio cholerae* O1 or enterotoxigenic *Escherichia coli* infection. Microbes Infect. (2011) 13:1111–20. doi: 10.1016/j.micinf.2011.06.014 21782033

[32] Vilander AC Hess A Abdo Z Ibrahim H Doumbia L Douyon S. A randomized controlled trial of dietary Rice bran intake on microbiota diversity, enteric dysfunction, and fecal secretory IgA in Malian and Nicaraguan infants. J Nutr. (2022) 152:1792–800. doi: 10.1093/jn/nxac087, PMID: 35441218 PMC9258582

[33] Lai H-H Chiu C-H Kong M-S Chang C-J Chen C-C. Probiotic *Lactobacillus casei*: effective for managing childhood diarrhea by altering gut microbiota and attenuating fecal inflammatory markers. Nutrients. (2019) 11:1150. doi: 10.3390/nu11051150, PMID: 31126062 PMC6566348

[34] Chen K Jin S Chen H Cao Y Dong X Li H. Dose effect of bovine lactoferrin fortification on diarrhea and respiratory tract infections in weaned infants with anemia: a randomized, controlled trial. Nutrition. (2021) 90:111288. doi: 10.1016/j.nut.2021.111288, PMID: 34102559

[35] Korpela K Salonen A Virta LJ Kumpu M Kekkonen RA de Vos WM. *Lactobacillus rhamnosus* GG intake modifies preschool Children's intestinal microbiota, alleviates penicillin-associated changes, and reduces antibiotic use. PLoS One. (2016) 11:e0154012. doi: 10.1371/journal.pone.0154012, PMID: 27111772 PMC4844131

[36] Rinne M Kalliomäki M Salminen S Isolauri E. Probiotic intervention in the first months of life. J Pediatr Gastroenterol Nutr. (2006) 43:200–5. doi: 10.1097/01.mpg.0000228106.91240.5b 16877985

[37] Linden J Koch-Nolte F Dahl G. Purine release, metabolism, and signaling in the inflammatory response. Annu Rev Immunol. (2019) 37:325–47. doi: 10.1146/annurev-immunol-051116-052406 30676821

[38] Bianco C Mohr I. Ribosome biogenesis restricts innate immune responses to virus infection and DNA. eLife. (2019) 8:e49551. doi: 10.7554/eLife.49551, PMID: 31841110 PMC6934380

[39] Ye Z Shi Y Lees-Miller SP Tainer JA. Function and molecular mechanism of the DNA damage response in immunity and Cancer immunotherapy. Front Immunol. (2021) 12:797880. doi: 10.3389/fimmu.2021.797880 34970273 PMC8712645

[40] Marquart ME. Pathogenicity and virulence ofStreptococcus pneumoniae: cutting to the chase on proteases. Virulence. (2021) 12:766–87. doi: 10.1080/21505594.2021.1889812, PMID: 33660565 PMC7939560

